# Biological Therapies in Oncology: Balancing Efficacy and Safety in the Context of Latent Tuberculosis

**DOI:** 10.34172/apb.43269

**Published:** 2024-10-02

**Authors:** Maher Monir. Akl, Amr Ahmed

**Affiliations:** ^1^Department of Chemistry, Faculty of Science, Mansoura University, 35516, Mansoura, Egypt.; ^2^The Public Health Department, Riyadh First Health Cluster, Ministry of Health, Saudia Arabia.

## Dear Editor,

 Cancer is a complex and multifaceted disease characterized by uncontrolled cell proliferation, invasion of surrounding tissues, and potential metastasis to distant organs. Among the various therapeutic approaches, biological therapy, or immunotherapy, has emerged as a groundbreaking modality.^1^ This treatment leverages the body’s immune system to recognize and combat cancer cells more effectively. Biological therapies include monoclonal antibodies, immune checkpoint inhibitors, cytokines, and cancer vaccines.^2^ For instance, monoclonal antibodies such as trastuzumab are used in the treatment of HER2-positive breast cancer, while immune checkpoint inhibitors like pembrolizumab are employed in managing melanoma and non-small cell lung cancer.^3^ The mechanism underlying cancer involves genetic mutations and epigenetic alterations that lead to the activation of oncogenes or the inactivation of tumor suppressor genes. These genetic changes disrupt normal cellular regulatory mechanisms, resulting in uncontrolled cell division and tumor formation.^4^ Additionally, cancer cells often evade immune surveillance by exploiting immune checkpoint pathways, making biological therapy a critical intervention by restoring the immune system’s ability to target and destroy malignant cells.^5^

 Latent tuberculosis infection (LTBI) is a condition wherein *Mycobacterium tuberculosis* resides in the body without causing active disease. Individuals with LTBI harbor the bacteria in a dormant state, typically within granulomas, and do not exhibit symptoms of tuberculosis nor can they transmit the infection to others.^6^ However, they remain at risk for developing active tuberculosis, particularly if their immune system becomes compromised. The pathogenesis of LTBI involves the inhalation of aerosolized droplets containing *M. tuberculosis*, which are then engulfed by alveolar macrophages in the lungs.^7^ In most cases, the host’s immune response successfully contains the bacteria within granulomas, preventing active disease manifestation. Nonetheless, a small percentage of those with LTBI will progress to active tuberculosis if the granulomas break down due to immunosuppression, malnutrition, or other health conditions.^8^

 Diagnosis of LTBI primarily relies on the Tuberculin Skin Test (TST) or Interferon-Gamma Release Assays (IGRAs), both of which detect immune sensitization to *M. tuberculosis* antigens.^9^ Treatment of LTBI aims to reduce the risk of progression to active tuberculosis and typically involves prolonged antibiotic therapy, such as isoniazid or rifapentine, often in combination with other agents.^10^ Biological therapies, also referred to as immunotherapies, have revolutionized cancer treatment by leveraging the body’s immune response to target and eradicate cancer cells. These therapies encompass a range of interventions, including monoclonal antibodies, immune checkpoint inhibitors, cytokines, and cancer vaccines.^11^ For instance, rituximab, a monoclonal antibody, selectively targets CD20 on B-cells in lymphomas, while nivolumab, an immune checkpoint inhibitor, blocks the PD-1 pathway, thereby enhancing T-cell response against tumors.^12^

 Despite their remarkable efficacy, these powerful immunotherapies carry the risk of inadvertently activating LTBI.^13^ The biological mechanisms underlying this phenomenon involve modulation of the immune system, particularly impacting T-cell function and cytokine production. For example, Pembrolizumab, an immune checkpoint inhibitor that inhibits PD-1 or PD-L1, stimulates a vigorous immune response by preventing T-cell exhaustion.^14^

 This heightened immune activity can disrupt granulomas containing dormant *M. tuberculosis*, leading to LTBI reactivation. Monoclonal antibodies like infliximab, which target tumor necrosis factor-alpha (TNF-α), are employed in specific cancers and autoimmune diseases.^15^ TNF-α plays a critical role in maintaining granuloma integrity, and inhibition of this cytokine can compromise granuloma structure, facilitating bacterial proliferation and potentially causing active tuberculosis.^16^

 Moreover, cytokines such as Interferon-gamma (IFN-γ) are essential for immune responses against both cancer and tuberculosis. Therapies modulating these cytokines may disturb immune homeostasis, inadvertently creating conditions conducive to LTBI reactivation.^17^ Thus, understanding the intricate immunological mechanisms involved is vital for optimizing the efficacy of cancer immunotherapy while mitigating the risk of LTBI reactivation. To mitigate these risks, it is crucial to implement screening protocols for LTBI before initiating biological therapy. The recommended diagnostic tests for LTBI are the TST and IGRAs. IGRAs, such as the Quanti FERON-TB Gold test, are particularly advantageous due to their higher specificity and not being affected by prior Bacille Calmette-Guérin (BCG) vaccination.^9^ The goal of implementing such screening protocols is to identify individuals with LTBI who are at risk of reactivation upon receiving biological therapy. By detecting and treating LTBI with appropriate prophylactic anti-tuberculosis medications, the risk of active tuberculosis can be significantly reduced, ensuring the safe administration of life-saving immunotherapies.

## Conclusion

 while biological therapies provide substantial advancements in cancer treatment, their potential to reactivate LTBI necessitates thorough pre-treatment screening. Implementing IGRAs for LTBI detection can effectively mitigate this risk, balancing the therapeutic benefits of immunotherapy with patient safety.

## Competing Interests

 The authors declare that there are no conflicts of interest.

## Ethical Approval

 Not applicable.
